# Comparison of health state utility estimates from instrument-based and vignette-based methods: a case study in kidney disease

**DOI:** 10.1186/s13104-019-4413-y

**Published:** 2019-07-08

**Authors:** Andrew H. Briggs, Vasily Belozeroff, David Feeny

**Affiliations:** 1 William R Lindsay Chair of Health Economics, Health Economics and Technology Assessment, Institute of Health & Wellbeing, University of Glasgow, 1 Lilybank Gardens, Glasgow, G12 8RZ Scotland UK; 2 Amgen Inc., Thousand Oaks, CA USA; 3 Department of Economics, McMaster University, Hamilton, ON Canada

**Keywords:** Health-related quality of life, Quality adjusted life years (QALYs), Cost-effectiveness, Medical technology, Health technology assessment (HTA)

## Abstract

**Objective:**

We take advantage of a rare occurrence when two different studies report on the estimation of quality of life utilities for the same health states to assess convergence of the reported measures. Health state utilities are important inputs into health economic models that estimate the impact of new medical technologies using a common metric of health gain—the quality adjusted life-year.

**Results:**

We find low concordance between the two measures which is concerning in that this could have important ramifications for health care decision making based on estimated cost-effectiveness. We explore possible reasons for the discrepancy between the two measures and draw implications for the design of future studies.

## Introduction

Quality adjusted life years (QALYs) are commonly used as a measure of overall health outcome in cost-utility analyses of medical technologies. QALYs combine life expectancy and quality of life in a common currency. Theoretical underpinnings of quality of life assessment suitable for estimating QALYs emphasize the need for a preference-based framework and contrast the implications of patients- vs. community-rated utilities [1–3]. In practice, when considering economic evaluation of medical therapies, sponsors are often confronted with the choice between the direct utility elicitation studies using standard gamble, visual analogue scale, time trade-off (TTO) approaches, and indirect utility elicitation in clinical trials based on value sets [4, 5]. Direct utility measures would involve development of health state descriptions (vignettes), while the indirect utility measures would employ a generic preference-based instrument, e.g., EQ-5D-3L, HUI or others as a measure in clinical trials [6–8]. Value sets are defined for a given instrument, such as the EQ-5D, as a method to translate questionnaires into utility values [9]. They are also based on an elicitation method, such as TTO, standard gamble, etc.

A recent report from an international task force offers considerations on utility assessment for healthcare technologies highlighting the need to align utility measurement with the health states of the corresponding economic analysis [10]. We report on a comparison of utility estimates for the same set of health states intended to inform a cost-utility model in the context of end stage renal disease (ESRD). These utilities were estimated using two different methods: the EQ-5D-3L instrument in a clinical trial, and a vignette-based TTO method in a general population. Our objective was to assess the general agreement among these methods using regression techniques, and to discuss the implications for cost-utility.

## Main text

### Summary of the studies

The first of the two studies was a randomized controlled clinical trial that evaluated the effects of a drug therapy cinacalcet on clinical outcomes, including cardiovascular events and fractures in patients treated for secondary hyperparathyroidism in end stage renal disease [11]. The EQ-5D-3L instrument was used in the trial at baseline and throughout a 5-year follow-up in over 3500 patients [6]. EQ-5D-3L health states associated with both acute (3 months post event) and chronic (3–12 months post event) were identified and valued using the UK-based EQ-5D-3L scoring system which is based on preference scores elicited using the TTO approach [12, 13].

The second study estimated the utilities of the same clinical events by surveying participants from the general population. The participants were presented with vignettes describing health states based on the inputs from focus group work with patients and clinical experts.

Health-state descriptions (vignettes) for health states associated with chronic kidney disease (CKD) and a parathyroid hormone condition, secondary hyperparathyroidism (SHPT), were developed based on literature review and a qualitative study involving 54 patients diagnosed with CKD and SHPT. The descriptions were then reviewed by three physicians who treat patients with CKD and SHPT. The description and methods for direct utility assessment using time tradeoff (TTO) were tested in a pilot study.

The vignettes described having CKD and SHPT with and without various cardiovascular or fracture outcomes both for the acute phase (the year during which the event occurred) and chronic phase (more than 1-year after the event). The vignettes described the disease, symptoms, the impact on physical and social activities, dialysis, and an event such as a heart attack. The time horizon for the TTO was 1 year. The TTO was administered in-person by a trained professional interviewer using the ping-pong approach and scored as x/y where x is the duration of time in perfect health that the subject values as being equal to spending the full year, y, in the impaired health state described in the vignette. 199 members of the general population (18+; mean age 46.3 years; 54.8% female; 49.7% reporting no health conditions) were interviewed; the group was not necessarily representative of the general adult population. Respondents were recruited through local newspaper (Toronto, Ontario, Canada) and online advertisements. Mean TTO scores and standard deviations were computed.

Study participants completed time trade-off interviews for both acute (a year including the event) and chronic (a year following the event) health states associated with clinical outcomes relevant to the same disease and treatment population [14].

### Results of the comparison

**Fig. 1:**
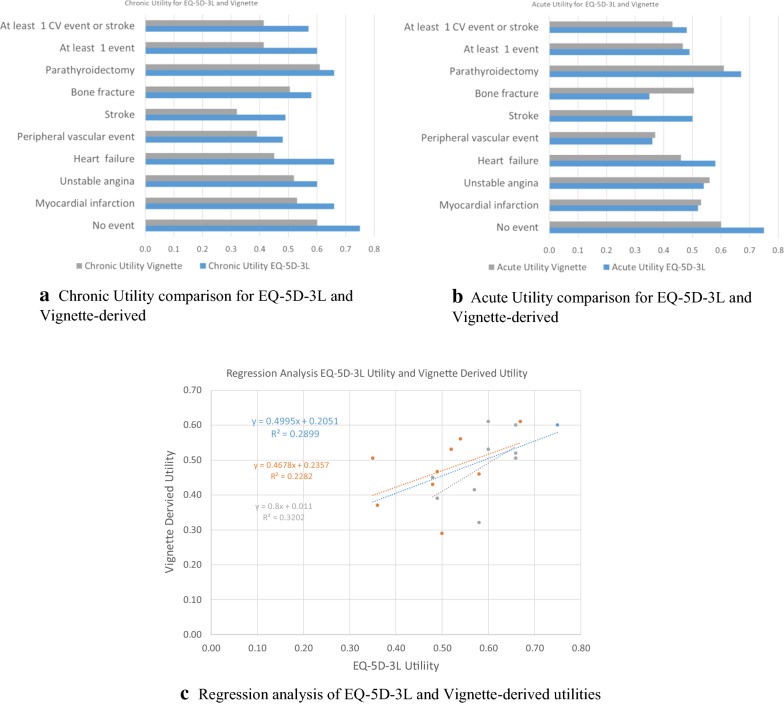
**a**–**c** Graphical comparison and regression analysis of EQ-5D-3L and Vignette-derived utilities

The comparison between the EQ-5D-3L derived utilities and the vignette-derived TTO utilities is presented in Fig. 1a, b). On average, utilities derived from EQ-5D-3L were higher than the utilities derived from the vignette study; average utility was 0.12 higher for EQ-5D-3L for chronic health states and 0.04 higher for acute health states. Several health states had excellent agreement across methods, namely, acute description of myocardial infarction, peripheral vascular disease, and hospitalized angina where the variation was within 4%. The event-free state had a major difference across methods (20%). Acute utility measures had a closer agreement than the chronic ones, with the exception of three states: Stroke, heart failure, and bone fracture (opposite direction). All chronic states were valued lower with the vignette-based method. An ordinary least squares (OLS) regression analysis of the two studies shows a 29% correlation between the EQ-5D-3L-derived and vignette-derived utility measures (Fig. 1c) for the overall data points. The correlation was somewhat stronger (R^2^ = 32%) if the regression was restricted to chronic compared to acute states (R^2^ = 23%), despite the larger average difference between the two studies for chronic states.

One of the greatest differences between the two studies was the ‘no event’ basic health state, which was 0.75 for the EQ-5D-3L based method, and 0.60 for the vignette-based method. An alternative comparison that could remove some of the inherent differences between the studies would be to look at the ‘disutility’ of the events estimated as the utility of the estimated event states less the basic no-event utility. Average differences in acute and chronic disutility for EQ-5D-3L and TTO settings was 0.07 but now with the EQ-5D-3L showing the higher disutility. An OLS regression analysis of the two studies for the disutility outcome shows a 23% correlation between EQ-5D-3L-derived and vignette-derived measures now that the baseline utility has been removed. Of course, restricting to acute or chronic events does not alter the correlation reported for utility above, although for disutility it is the acute events that have the largest magnitude of difference between the measures.

## Discussion

We compared utility estimates from two well-accepted methods assessing health state utilities to inform economic analysis of medical interventions. Both the vignette-based and the instrument-based methods are theoretically correct, but the evidence is limited as to their convergence. An earlier review of health utility measures and elicitation noted that study population characteristics such as socioeconomic demographics and disease or health states should be taken into consideration when deriving health utilities from vignettes [15]. Another study comparing community quality of well-being (QWB) and patient-based preferences (TTO) for health outcomes from randomized controlled trial data found that QWB preference scores were significantly lower than corresponding TTO scores, which may have important implications for estimating QALYs gained [16].

Utility score estimates derived from members of the general population may help approximate a societal viewpoint, but the utility assessment involving a hypothetical health state is limited by the accuracy and level of detail in the health state descriptions. Additionally, including a label of the health state being described and the setting in which the vignettes are presented (online or in-person) may affect the respondents’ valuations of the state [17, 18]. Direct utility elicitation using the TTO (i.e., based on vignettes) is considered appropriate/acceptable by some HTA bodies when EQ-5D-3L is not available, and TTO with a 10-year time horizon is the most frequently used approach among the direct techniques, because of greater comparability with the method used to develop the EQ-5D-3L scoring algorithm [19, 20].

Switching to disutility has a potentially important effect on both the estimated impact of clinical events and on cost-effectiveness. The calculation of disutility emphasizes the impact of clinical events and the importance of the baseline utility to which they are applied. In comparing the two studies, the large difference between the basic health state utility without the event was enough to reverse the magnitude of the differences between the studies. Utilities look higher when using the EQ-5D-3L instrument, but that disutility of events was also higher due to the higher no-event utility.

We found a weak positive correlation between both the chronic and acute utility regressions (Fig. 1c). This means there is substantial unexplained variability demonstrated between the studies that are measuring the same health states, and that mapping between the two can only be weakly predicted. This is concerning as such large utility differences may impact QALY estimates when calculating cost effectiveness of drugs and other interventions. Thus, differences in utility methodologies may lead to variabilities in ICER calculations when using EQ-5D-3L and vignette methods.

As there is no gold standard for utility measurement, our goal was not to “validate” a method but rather to compare, and we found that the methods were only weakly correlated. Importantly, there were no *consistent* differences other than the utility being lower for chronic sub-states for vignette-based method, which may reflect patient adaptation [10].

From a practical standpoint, it is reasonable to use a method that would be accepted by a specific decision-maker. For instance, in a recent assessment of a novel therapy in end stage renal disease, the UK National Institute for Health & Care Excellence (NICE) had accepted the EQ-5D-3L based utilities in a cost-utility model [21]. If a clinical trial with a reasonable duration and health state measurement opportunity is being planned, then including an instrument in such a trial is a good strategy—longitudinal data allow application of regression models to estimate health state utilities. Where such trials are not available, or have already been completed without a utility instrument, a bespoke study to collect utilities seems the only option. A vignette-based method would allow a detailed description of the health states, but will invariably introduce a level of subjectivity. This same situation may arise where valuation of acute health states is wanted but may not be practically assessed in actual patients. To reduce the level of subjectivity, one could argue that a standard battery of vignettes ought to be catalogued so research is consistent across studies.

There are some nuanced differences that one could describe as “you get what you measure” and “you get what you describe” for each of the methods. In other words, if one is able to include multiple EQ-5D-3L measures in a trial, then there are opportunities to explore the data in a way that is fitting an economic model. Similarly, one can describe details in the vignettes to the level that helps discern details important for patients but not visible through a generic instrument, in time as well as in content. As a contrast, limited measurements of an instrument in a trial, like limited descriptions within vignettes, would hamper such flexibility on either side.

We recognize that the value set from Dolan et al. has been heavily criticized in the past and has several issues. One particular issue is the use of a linear regression model for utility values [22]. Authorities such as NICE should consider the criticism of existing value sets, creating more appropriate ones, and provide strong guidance on which value sets to use. Furthermore, the findings of this analysis could suggest that it might be important that all utility values should be quantified based on a consistent approach.

The comparison of the health states utilities in these two studies lies within a general realm of health states whose consequences would be fairly well understood and appreciated by the medical community and the general population when described as vignettes. In that sense, one would expect that the real experience of a health state measured by an instrument in patients would be comparable to a hypothetical imaginary experience by non-patients based on a description of this state, barring the adaptation aspect. A more nuanced health state that requires more details and that is not as readily known to a non-patient may call for a different approach, but that would challenge both vignette-based methods on the ground of complexity and added subjectivity, and the instrument-based approach on the grounds of sensitivity. These are important considerations for further research and methodological harmonization.

## Limitations

It is important to note that there are nuances in the temporal characteristics of the state definitions that may impact scores. There have been other analyses comparing different ways of estimating utility values, and it is well known that different approaches may yield different values [23–25]. There may be more than one factor that contributes to the difference between two utility scores. These differences include but are not limited to instrument vs. direct method, nuances in temporal definitions of the states, differences in the inclusion of events by severity (which may explain, for example, the difference in fracture utilities in our comparison), and differences in the general population vs. actual patients. In this paper, we use a generic (non-specific) measure, the EQ-5D-3L, whereas the direct elicitation method is more disease specific.

## Data Availability

All data generated or analysed during this study are included in this published article.

## Abbreviations

QALY: quality adjusted life year
TTO: time trade-off
ESRD: end stage renal disease
SHPT: secondary hyperparathyroidism
QWB: quality of well-being
